# Vancomycin-Resistant Staphylococcus Aureus Infection Post-Liposuction in South Korea

**DOI:** 10.7759/cureus.14357

**Published:** 2021-04-07

**Authors:** Erni J Nelwan, Dewi Andayani, Gabriella Clarissa, Trisoma Pramada

**Affiliations:** 1 Division of Tropic and Infectious Disease, Department of Internal Medicine, Universitas Indonesia, Jakarta, IDN; 2 Faculty of Medicine, Universitas Katolik Indonesia Atma Jaya, Jakarta, IDN; 3 Department of Surgery, Metropolitan Medical Centre Hospital, Jakarta, IDN

**Keywords:** medical tourism, vrsa, antibiotic resistant

## Abstract

As antibiotic resistance becomes a serious health issue, medical tourism is an accelerating factor. Several studies report antibiotic-resistant cases in Southeast Asia are increasing every year. We report the first case of a vancomycin-resistant *Staphylococcus aureus* (VRSA) infection in an Indonesian post-liposuction in South Korea. The patient is a 34-year-old Indonesian woman reporting concerns of fever and abdominal abscess post-liposuction. Culture results before antibiotic therapy were positive for VRSA. After the patient received one-time abscess drainage and initiated oral broad-spectrum antibiotics, the abscess clinically improved. To this date, the most common complication of infection post-liposuction in Indonesia is related to Mycobacterium as etiology. The pathogen transfer correlates to medical tourism, and this becomes a reminder for health care providers to be prepared to encounter problems tied to medical tourism.

## Introduction

Antibiotic resistance is becoming an increasingly worrying health problem, especially given the development of medical tourism, which increases barriers to the transfer of bacterial pathogens across regions [1,2]. One study reported the most common etiology of infection post-liposuction is related to Mycobacterium [3]. For Southeast Asians, South Korea is a favorite destination for cosmetic surgery [4]. The concern is South Korea has the highest levels of methicillin-resistant *Staphylococcus aureus* (MRSA) and vancomycin-resistant Enterococcus (VRE) in the Regional Resistance Surveillance (RRS) study programs, so physicians need to be more vigilant in treating patients who have recently undergone procedures abroad [5].

## Case presentation

**Figure 1:**
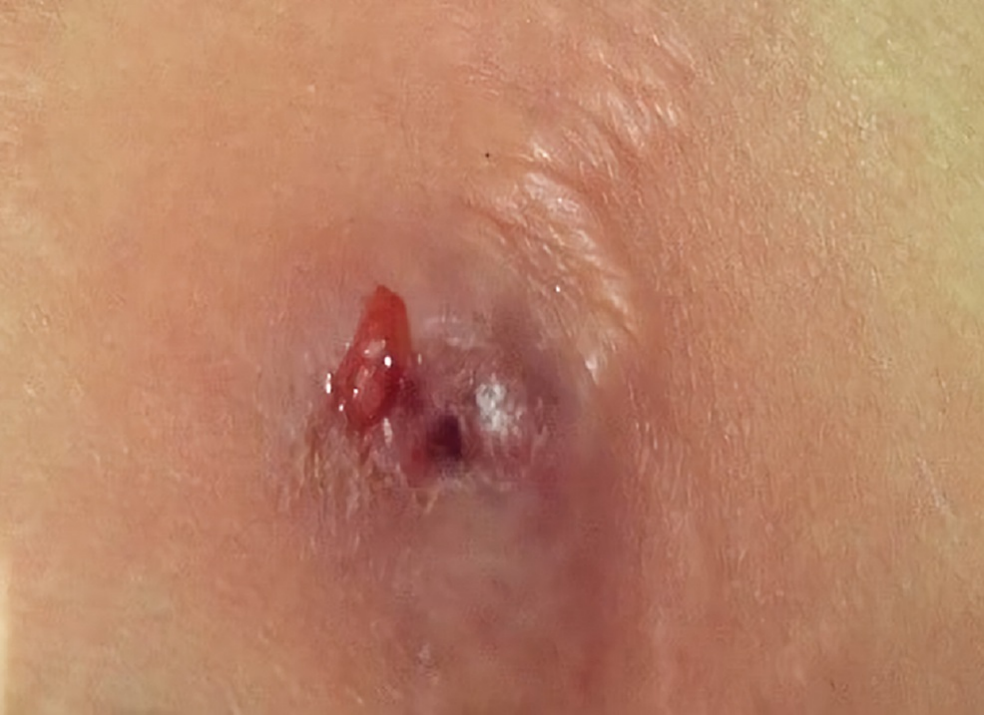
Image of the liposuction site on initial presentation revealing a ruptured abscess and minimal systemic sign

A 34-year-old Indonesian woman reported concerns of fever and an abscess at the site of liposuction three days before coming to the outpatient of infectious disease (ID) clinic. She received liposuction in South Korea three weeks before the consultation. The procedure was one-day surgery, thus after the surgery was done patient was discharged from the hospital and continued her activity daily living. Several days after, the patient realized that the surgical wound has not completely healed since the date of the procedure and decided to went to the ID clinic, there is no history of taking the drug without any prescription. Physical examination of the abdomen revealed a ruptured abscess and minimal systemic sign (Figure 1). Laboratory results showed leukocytosis (15,000/mm^3^), increased neutrophil count (92%), increased C-reactive protein levels (78mg/L), and decreased procalcitonin levels (1ng/mL).

**Figure 2:**
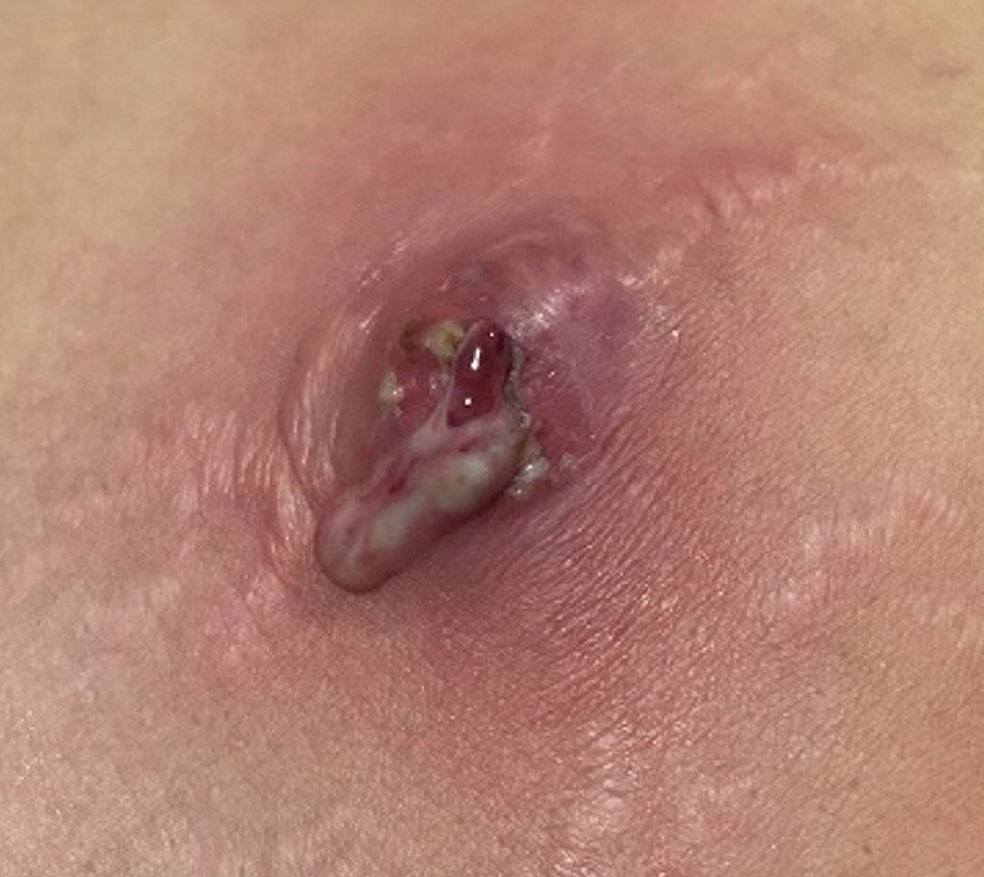
Image of the liposuction site after three days of antibiotic treatment, prior to surgical drainage

Considering the possibility of a gram-positive bacterial infection, we initially administered clindamycin and moxifloxacin as broad-spectrum antibiotics to cover the pathogens of Staphylococcus and Mycobacterium empirically. The patient was started on oral clindamycin (300mg, four times a day) and oral moxifloxacin (400mg, once a day). On day 3, an ID physician evaluated and diagnosed the patient with acute bacterial skin and skin structured infection, the ID physician recommended she undergo surgery for abscess drainage (Figure 2). A pus sample was taken for *Mycobacterium *culture.

**Figure 3:**
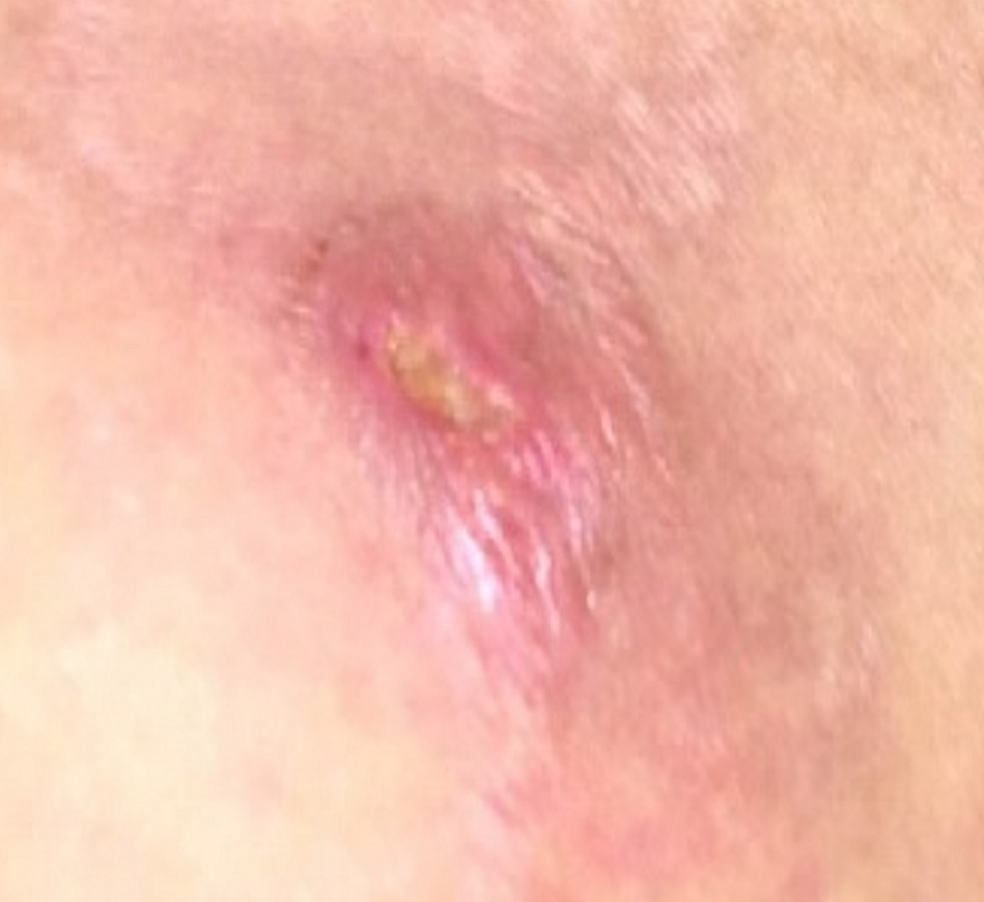
Image of the liposuction site after surgical drainage on the fourth day of antibiotic treatment

**Figure 4:**
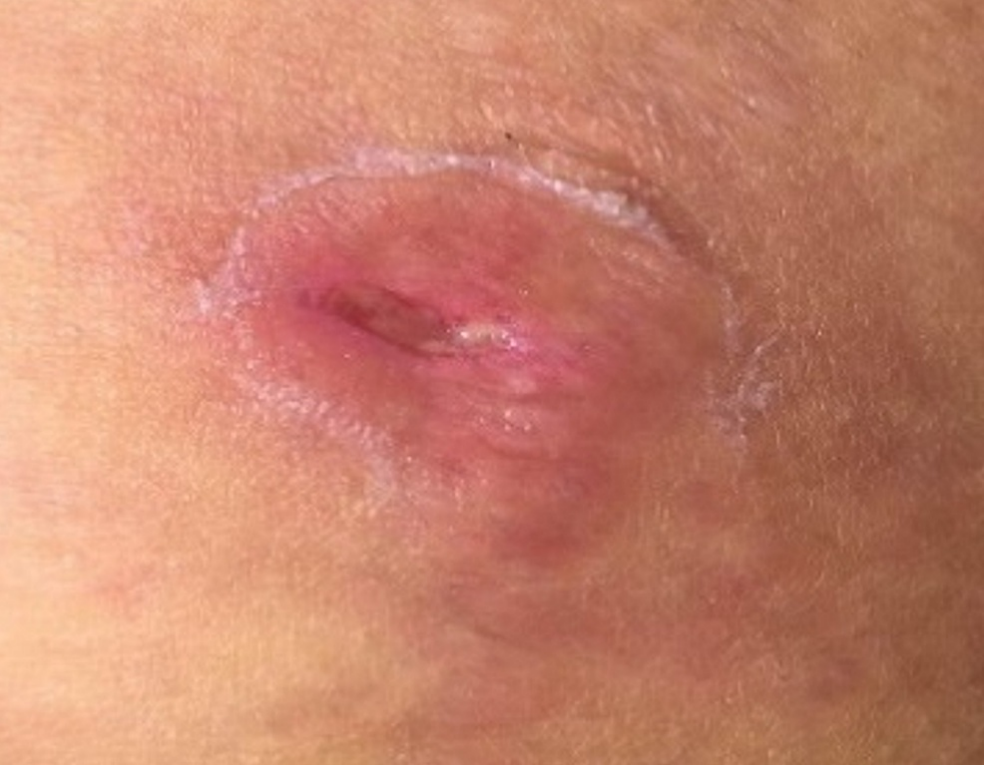
Image of the liposuction site on the seventh day of antibiotic treatment

**Figure 5:**
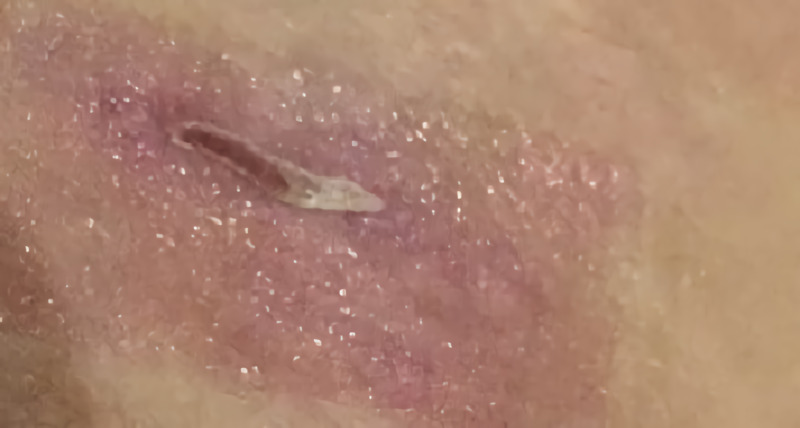
Image of the liposuction site on the tenth day of antibiotic treatment.

After the patient received one-time abscess drainage and was initiated with clindamycin and moxifloxacin, the abscess clinically improved (Figure 3). The patient got an evaluation on day seven and day 10 after antibiotics treatment and antibiotics were continued for two weeks (Figures 4, 5). 

The result from the culture performed before the antibiotics treatment revealed vancomycin-resistant *S. aureus. *

## Discussion

Medical tourism refers to patients traveling abroad to get medical treatment either not available or more affordable than what is available in their home country. Information is easily available given the development of electronic media, and the ease of international travel has encouraged the growth of this industry [6]. In addition to the benefit, physicians and patients should be aware of the concurrent risks of medical tourism. Immunocompromised and immunodeficient patients have a higher risk of coming down to infectious diseases. The increasing popularity of medical tourism-especially for cosmetic surgical procedures performed in foreign countries has led to a rise in the incidence of non-tuberculosis mycobacteria (NTM) infections [7].

The current popularity of medical tourism is largely due to five factors: affordability, accessibility, availability, acceptability, and additional care. Availability refers to whether the medical treatment is offered in the patient’s home country or not. Accessibility refers to the immediacy of the procedures. Acceptability refers to services that available, affordable, and accessible but not acceptable in a patient’s home country for societal, social, or religious reasons. Additional care is the availability of better care service including better technology and better provider care abroad compared to that offered in the patient’s home country [8]. 

One popular destination for Southeast Asians to undergo cosmetic surgery is South Korea. However, the latest report from the RRS indicates that, among 12 countries, South Korea has the highest MRSA rate (73%) and VRE rate (26%) with the vancomycin A gene (vanA gene; 80%) compared to Indonesia which has an MRSA rate of 28% and a VRE rate of 0% (i.e., undetected) [5,9]. Hypothetically, VRSA related to the vanA gene integrated into a plasmid may originate from VRE.

One study claimed the most common complication post-cosmetic surgery is wound infection (26%) at the procedure site [10]. Incision and pus/debris drainage are the mainstay of skin abscess treatment [9,11]. *S. aureus* is the leading cause of skin and soft tissue infections, osteoarticular, pleuropulmonary, bacteremia, and infective endocarditis [12]. Delaying the antibiotic administration until the results of the culture test are available may double the risk of serious infection associated with *S. aureus*. Therefore, early initiation of antibiotic drugs and surgical drainage of the abscess are key elements in overall management.

## Conclusions

The first documented case of VRSA in Indonesia illustrates how the pathogen transfer occurs and should remind health care providers to be prepared for similar problems. Due to a lack of data, more studies are needed to examine the negative consequences of medical tourism along with the potential benefits. It is hoped that this case presentation will further increase the awareness of the healthcare provider in order to reduce the pathogen transfer due to medical tourism.
